# Mitochondria and Cytoprotection

**DOI:** 10.1155/2012/351264

**Published:** 2012-08-16

**Authors:** Catherine Brenner, Renée Ventura-Clapier, Etienne Jacotot

**Affiliations:** ^1^Inserm UMR-S 769, LabEx LERMIT, Châtenay-Malabry, F-92296, France; ^2^University Paris-Sud, Châtenay-Malabry, F-92296, France; ^3^Inserm UMR 676, Hôspital Robert Debré, F-75019 Paris, France; ^4^Faculté de Médecine Denis Diderot, Université Paris 7, IFR02, F-75019 Paris, France; ^5^Faculty of Medicine, Institute of Reproductive and Developmental Biology, Hammersmith Hospital, Imperial College London, London W12 0NN, UK

Mitochondrion is an eukaryotic double-membrane bound organelle that arose from a bacterial endosymbiont. Its circular DNA is small, as many genes were transferred to nucleus during evolution, and almost exclusively maternally inherited. Mitochondria harbor highly regulated biogenesis activities (division and fusion) and motility, establish interorganellar contacts, and perform/house multiple and interconnected cellular functions, as diverse as ATP production (via oxidative phosphorylation), parts of pyrimidine and lipid biosynthetic pathways (including the fatty acid *β*-oxidation pathway), reactive oxygen species production and detoxification, and metal metabolism (synthesizing heme and Fe–S clusters). Mitochondria also participate in calcium homeostasis, metabolic sensing, innate immunity, autophagy, and cell death regulation. The coordination of these functions contributes to the cell and life balance and mitochondrial dysfunction is consequently associated with many human diseases. 

On the one hand, discovery of the major role of mitochondria in cell death signaling and fate has led to important conceptual changes in oncology and has enriched anticancer strategies. On the other hand, structurally and functionally abnormal mitochondria have been described in nonalcoholic fatty liver diseases, as well as many neurodegenerative diseases including Alzheimer's disease, Parkinson's disease, amyotrophic lateral sclerosis, and Huntington's disease. Mitochondrial failure has been shown to be central to ischemia/reperfusion damages. An energetic imbalance involving impaired mitochondrial respiration and substrate utilization has been demonstrated in cardiopathies in rodents as well as in human. 

Increasing evidence indicates that cytoprotection via preservation of mitochondrial function is a promising therapeutic strategy to limit the severity of the diseases and their progression. In this special issue, we have invited reviews that discuss the interest and the modalities of cytoprotection in diverse conditions or experimental settings. 

At the crossroad of physiology, metabolism, and toxicology, liver mitochondria have been intensively studied. Their particular importance in chronic liver disease is discussed by D. Esposti and coworkers. Mitochondria also play a key role in glucose-stimulated insulin secretion. Human pancreas islet transplantation is a promising therapy for type I diabetes mellitus. However, innovative methods are required for *β*-cell preservation against oxidative stress, inflammation, and cell death signaling. Y. Wang et al. present a detailed review of the recent progress in mitochondrial cytoprotection in each step of the islet isolation and transplantation process.

Prevention of mitochondrial membrane permeabilization to block cell death is a powerful concept, with many therapeutic implications in organoprotection (e.g., heart, brain, liver, and kidneys). In a number of acute conditions like ischemia-reperfusion, the so-called mitochondrial permeability transition (MPT) is an operational checkpoint to cell death. C. Martel et al. discuss how MPT is regulated and can be manipulated to reduce cell death. 

The clinical use of anthracyclines in anticancer therapies is severely limited by the development of a progressive dose-dependent cardiomyopathy that irreversibly evolves toward congestive heart failure. D. Montaigne and coworkers review cytoprotection strategies to reduce the cardiotoxicity of anthracyclines.

The paper by J. Kang and S. Pervaiz addresses the central issue of redox metabolism, and the role of mitochondria as regulators of redox homeostasis and cell fate. They describe how dysfunction of mitochondria and the ensuring oxidative stress is one of the causal factors of a variety of diseases including neurodegenerative diseases, diabetes, cardiovascular diseases, and cancer. From the current understanding of reactive oxygen/nitrogen species generation and regulation in the mitochondria and of their impact on cell fate, the authors propose some clues on how redox metabolism could be targeted for therapeutic purposes. 

Oxydative stress, mitochondrial signaling, and dysfunction can also be induced through exposure to pollution particles (for instance generated by combustion of wood or diesel oil) and to gaseous ligands capable of binding to either hemoglobin or mitochondrial cytochrome *c* oxidase (e.g., NO, CO, and H_2_S). K. Andreau et al. discuss how air pollutants impact redox status, metabolic, and apoptotic functions of mitochondria, and thereby have major consequences on cell death and health, with societal burden. Finally, C. S. F. Queiroga et al., focused on carbon monoxide, a toxic gas, which can be used, however, as a therapeutic agent.

In conclusion, this special issue discusses some aspects of cytoprotection through mitochondrial function preservation. Several of these reviews and our own past and present research suggest that this strategy might help to delineate efficient preventive or therapeutic strategies.

*Catherine Brenner* *Renée Ventura-Clapier* *Etienne Jacotot*

